# Increased Levels of Plasma Alzheimer’s Disease Biomarkers and Their Associations with Brain Structural Changes and Carotid Intima-Media Thickness in Cognitively Normal Obstructive Sleep Apnea Patients

**DOI:** 10.3390/diagnostics12071522

**Published:** 2022-06-22

**Authors:** Yueh-Sheng Chen, Meng-Hsiang Chen, Pei-Ming Wang, Cheng-Hsien Lu, Hsiu-Ling Chen, Wei-Che Lin

**Affiliations:** 1 Department of Diagnostic Radiology, Kaohsiung Chang Gung Memorial Hospital, Chang Gung University College of Medicine, Kaohsiung 833, Taiwan; yssamchen@gmail.com (Y.-S.C.); sperfect@msn.com (M.-H.C.); 2 Department of Family Medicine, Kaohsiung Chang Gung Memorial Hospital, Kaohsiung 833, Taiwan; wangpeming@yahoo.com.tw; 3 Department of Neurology, Kaohsiung Chang Gung Memorial Hospital, Chang Gung University College of Medicine, Kaohsiung 833, Taiwan; chlu99@ms44.url.com.tw

**Keywords:** obstructive sleep apnea, Alzheimer’s disease, biomarker, neuroimaging

## Abstract

Obstructive sleep apnea (OSA) has been linked to Alzheimer’s disease (AD) and amyloid deposition in the brain. OSA is further linked to the development of cardiovascular and cerebrovascular diseases. In this study, we analyzed the plasma levels of AD neuropathology biomarkers and their relationships with structural changes of the brain and atherosclerosis. Thirty OSA patients with normal cognition and 34 normal controls were enrolled. Cognitive functions were assessed by the Wechsler Adult Intelligence Scale third edition and Cognitive Ability Screening Instrument. Plasma Aβ-40, Aβ-42, and T-tau levels were assayed using immunomagnetic reduction. The carotid intima-media thickness was measured to assess the severity of atherosclerosis. Structural MR images of brain were acquired with voxel-based morphometric analysis of T1 structural images. The OSA patients exhibited significantly elevated plasma levels of Aβ-42 and T-tau, as well as increased gray matter volume in the right precuneus. Plasma T-tau level is associated with carotid intima-media thickness and gray matter volume of the precuneus. These findings may indicate early changes that precede clinically apparent cognitive impairment. The measurement of these biomarkers may aid in the early detection of OSA-associated morbidity and possible treatment planning for the prevention of irreversible neuronal damage and cognitive dysfunction.

## 1. Introduction

Obstructive sleep apnea (OSA) is a common breathing disorder during sleep which is associated with intermittent hypoxia and sleep fragmentation [1]. OSA is associated with Alzheimer’s disease (AD) and amyloid deposition in the brain [2,3]. Amyloid β (Aβ) aggregation with plaque development and tau-hyperphosphorylation forming neurofibrillary tangles are hallmarks of AD neuropathology [4]. The possible mechanisms responsible for AD neuropathology in OSA patients include intermittent hypoxia, sleep fragmentation, and elevated intrathoracic and intracranial pressures [5]. Currently, methods for assessing AD neuropathology in a clinical setting include positron electron tomography (PET) imaging, cerebrospinal fluid (CSF)-based analysis, and blood-based analysis. Among these, blood-based analysis is less invasive and more cost-effective for routine clinical use [6].

As normal functioning of the sleep-wake cycle is necessary for regulating the clearance of amyloid, the characteristic sleep disruption in OSA patients may impair such clearance, resulting in the development of AD neuropathology. Furthermore, vascular injury is an important factor in the development of Aβ plaque pathology, possibly due to the impaired neuronal supply of nutrients and reduced Aβ clearance [4]. In OSA patients, there is considerable evidence of its association with the development of cardiovascular and cerebrovascular diseases [7,8]. Carotid intima-media thickness (IMT), a valuable marker for atherosclerosis, has been investigated for its association with OSA patients [9,10]. The presence of increased oxidative stress and inflammation in OSA patients may contribute to vessel injury and the development of atherosclerotic diseases [9]. Meanwhile, vascular damage may also contribute to the development of AD neuropathology and cognitive impairment [11].

Magnetic resonance imaging (MRI) is an effective tool for investigating changes of the brain non-invasively. Voxel-based morphometry (VBM) is an assumption-free, objective, data-driven approach for evaluating gray matter volume (GMV) differences in a voxel-wise manner throughout the brain. Previous studies have reported inconsistent findings with regards to the potential link between structural and functional brain changes in OSA patients. More specifically, studies have reported both increased and decreased GMV and functional connectivity in various brain regions in OSA groups as compared to normal controls [12,13].

In this study, we investigated the hypothesis that OSA patients would exhibit increased plasma levels of AD neuropathology biomarkers. We also explored the relationships among these biomarkers, IMT, and structural changes of the brain. By studying patients with preserved cognitive function, our results may indicate early changes prior to the occurrence of irreversible brain damage and cognitive impairment.

## 2. Materials and Methods

### 2.1. Subjects

This retrospective cross-sectional study consisted of 30 OSA patients and 34 age-matched normal controls (NCs). The OSA patients and 17 of the NCs were recruited from the Kaohsiung Chang Gung Memorial Hospital Sleep Center with a primary complaint of snoring. Another 17 NCs were enrolled from the community without sleep-related symptoms. All participants with snoring had an overnight polysomnography (PSG) examination and were divided into patient and control groups based on the standard diagnostic criteria [14]. The definition for apnea/hypopnea index (AHI) was the number of apneas and hypopneas per hour in the sleep time. The diagnosis of OSA was made if the patients have OSA symptoms while having an AHI of more than 5/h or those solely having an AHI of more than 15/h. We excluded participants with central type sleep apnea. None of the participants had received OSA-related treatment. We excluded all participants with major neurological or psychiatric disorders, head trauma, stroke, diabetes mellitus, obesity (BMI ≥ 30), and major cardiovascular disorders. The Kaohsiung Chang Gung Memorial Hospital Ethics Committee approved this study.

### 2.2. Neuropsychological Assessments and Carotid IMT Measurement

The neuropsychological testing included subtests from the Chinese versions of the Wechsler Adult Intelligence Scale-III (WAIS-III) [15] and the Cognitive Ability Screening Instrument (CASI) [16], which were performed by a clinical psychologist who was blinded to the grouping status of each participant. The attention function was assessed by the digit span test from WAIS-III, and the attention and orientation tests from CASI. The executive function was assessed by the digit symbol coding and arithmetic tests from WAIS-III, and the abstract thinking test from CASI. The memory function was assessed by the short-term and long-term memory tests from WAIS-III, and the information test from CASI. The speech and language function was assessed by the comprehension test from WAIS-III, and the language and semantic fluency tests from CASI. The visuospatial function was assessed by the picture completion and block design tests from WAIS-III, and the drawing test from CASI.

IMT is defined by the distance between the intima–blood interface and the adventitia–media junction. The carotid IMT measurement was conducted in accordance with a previous report [9]. The extracranial color-coded duplex sonography (ECCS) examination was conducted using the B-mode ultrasound system (Philips HDI 5000 System, 4–10 MHz linear array transducer; Advanced Technology Laboratories-Philips, Bothell, WA, Australia). The images were obtained at the common carotid artery in the longitudinal plane along a 1-cm length proximal to the carotid bulb. The measurement was done automatically by the QLAB quantification software (ATL-Philips). All IMT measurements were performed by the same experienced sonographer, who was blinded to the participants’ condition, by measuring both sides of the common carotid artery and reporting the average.

### 2.3. Blood Samples and Assessment of Plasma AD Biomarkers (Aβ-40, Aβ-42, T-tau)

All participants received a forearm vein venipuncture between 10:00 a.m.–11:30 a.m. to avoid potential interference due to the sleep-wake cycle. Blood samples were collected using a 10-mL K3-EDTA tube (Greiner Bio-One 455036) and centrifugation was conducted at 1500–2500× *g* at a temperature of 15–25 °C for 15 min using a swing-out rotor. Then, 0.5 mL of plasma was removed from the blood tube and transferred into a fresh 1.5 mL Eppendorf tube. Prior to performing the assays, the aliquoted plasma samples were frozen at a temperature of −80 °C within 3 h of the blood draw.

The analysis of AD biomarkers using immunomagnetic reduction (IMR) technology has been described in previous reports [17,18]. Various kits for assaying the concentrations of AD biomarkers consisting of magnetic Fe_3_O_4_ nanoparticles reagents (total tau (T-tau) (MF-TAU-0060, MagQu), Aβ-40 (MF-AB0-0060, MagQu), Aβ-42 (MF-AB2-0060, MagQu)) that were biofunctionalized with monoclonal antibodies (sc-12767, Santa Cruz Biotech) were used. Eighty microliters of reagents were mixed with 40 µL of plasma for assaying the concentrations of T-tau and Aβ-40. Sixty microliters of reagents were mixed with 60 µL of plasma for assaying the concentration of Aβ-42. The IMR signals from the magnetic concentrations of the immunocomplex were detected using a superconducting-quantum-interference-device (SQUID)-based alternative current (ac) magnetosusceptometer (XacPro-S, MagQu). The signals were then transformed into biomarker concentrations via the concentration-dependent IMR signal. A duplicate measurement was performed for each biomarker testing. The detected concentrations were determined by the average value of the duplicated measurements. The coefficient of variation (CV), defined as standard deviation divided by average, was used for quality control. A CV below 20% was considered acceptable. If a CV of greater than 20% occurred, we performed an additional measurement. We selected two of the three measurements resulting in a CV below 20% for averaging to achieve the final concentration of the biomarker. The limit of detection in Tau, Ab40 and Ab42 with IMR are 0.026, 0.53, and 0.77 pg/mL, respectively [19,20].

### 2.4. MRI Data Acquisition

#### Image Acquisition

The MRI scan was performed using a 3.0 Tesla whole-body GE Signa MRI system (General Electric Healthcare, Milwaukee, WI, USA) equipped with an eight-channel head coil. Each participant’s head was immobilized with foam pillows in the coil to minimize motion. The T1-weighted image was acquired along the AC-PC line using the three-dimensional fluid-attenuated inversion-recovery fast spoiled gradient recalled echo sequence (3D IR-FSPGR, repetition time(TR)/echo time(TE)/inversion time = 9.5/3.9/450 ms, flip angle = 20°, FOV = 25.6 cm, matrix size = 512 × 512, voxel size = 0.47 × 0.47 × 1.3 mm^3^, 110 slices without gaping). An experienced neuroradiologist examined all images to check for the presence of any brain abnormalities.

### 2.5. Image Preprocessing

The images were preprocessed by Statistical Parametric Mapping 12 (SPM12; University College London) running on Matlab R2016a (Mathworks). The T1-weighted structural MR images were segmented into gray matter (GM), white matter (WM), and cerebrospinal fluid (CSF) compartments during the segmentation process. The DARTEL (Diffeomorphic Anatomical Registration Through Exponentiated Lie Algebra) algorithm was applied for the normalization process. The study-specific tissue templates were created using MR images from all participants. The GM segments were warped into the new reference space with interpolation to 1.5 mm isotropic voxels. Modulation was performed to preserve the actual volumetric information, and the warped GM segments were affine transformed into Montreal Neurological Institute (MNI) space. Smoothing was conducted on the modulated GM segments using an 8-mm full-width-at-half-maximum (FWHM) Gaussian kernel. The probability threshold was set at 0.2 to avoid an edge effect for possible incorporation of tissue with lower GM probability. Total intracranial volumes (TIV) were calculated by adding up the total voxels of GM, WM, and CSF in the native space separately.

### 2.6. Statistical Analyses

#### 2.6.1. Analyses of Demographic Data, Plasma Biomarkers, Clinical Assessments, and Neuropsychological Testing

Age and BMI data were analyzed using independent t tests, while sex data were compared using the Pearson chi-square test. The plasma biomarkers, polysomnography parameters, and IMT of CCA were compared by analysis of covariance (ANCOVA) after controlling for age and sex. The neuropsychological testing scores were compared by ANCOVA after controlling for age, sex, and education level. Only tests where OSA patients had significantly different scores than controls were considered for correlation analysis. Correlations between plasma biomarkers, GMV, and clinical assessments were conducted by partial correlation analysis with age and sex as covariates. Continuous variables were presented as mean ± standard deviation. Statistical analysis was performed using the Statistical Product and Service Solutions software version 19 (IBM SPSS). A *p* value less than 0.05 was considered statistically significant.

#### 2.6.2. Voxel-Based Morphometry Analysis

The GMV comparison between OSA patients and normal controls was performed using voxel-wise group comparisons with full factorial design. Age, sex, and total intracranial volume (TIV: calculated as the sum of the total voxels of GM, WM, and CSF) were included as covariates. This facilitated the detection of any regional GMV differences that may exist between the two groups. The results were considered significant under the criteria of family-wise error (FWE)-corrected *p* value < 0.05, using Monte Carlo simulation with a cluster size of at least 228 voxels (AFNI 3dClusterSim with the following parameters: single voxel *p* value < 0.001, full width at half maximum (FWHM) = 8 mm with gray matter mask, and 10,000 simulations).

## 3. Results

### 3.1. Baseline Clinical Characteristics of OSA Patients and Controls

The baseline clinical demographic data and neuro-psychological assessment scores of all subjects are shown in Table 1. The OSA group had significantly more males than the control group. There were no significant age or BMI differences between the OSA patients and controls. As expected, there were significant differences in the polysomnography parameters and IMT of CCA between the OSA patients and controls. None of the subtests in all five domains of NPT showed significant differences between the two groups.

### 3.2. Plasma Biomarkers between OSA Patients and Controls

**Figure 1:**
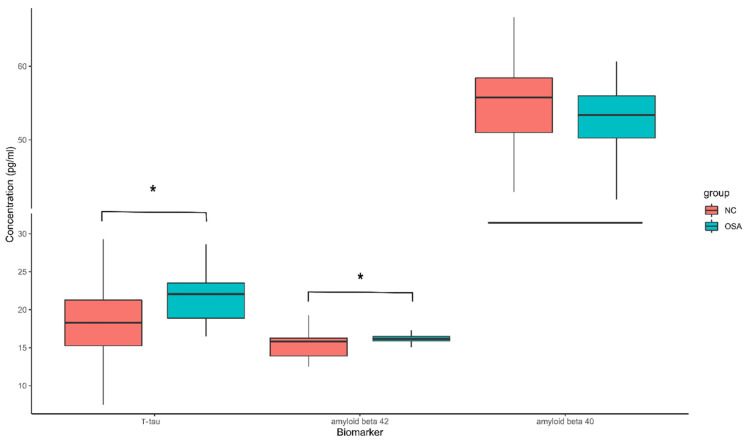
The boxplot shows the plasma levels of AD biomarkers. The OSA group had significantly higher levels of plasma T-tau and Aβ42. The Aβ40 level showed no significant difference between the two groups. * *p* < 0.05.

The levels of plasma biomarkers were analyzed using IMR. The OSA patients had significantly higher plasma T-tau (21.43 ± 0.55 pg/mL in OSA patients; 18.27 ± 0.85 pg/mL in normal controls) and Aβ42 (16.17 ± 0.12 pg/mL in OSA patients; 15.37 ± 0.26 pg/mL in normal controls). Aβ40 (53.00 ± 0.90 pg/mL in OSA patients; 54.61 ± 1.25 pg/mL in normal controls) showed no significant difference between the two groups (Figure 1).

### 3.3. Comparison of Regional GMV between OSA Patients and Controls

**Figure 2:**
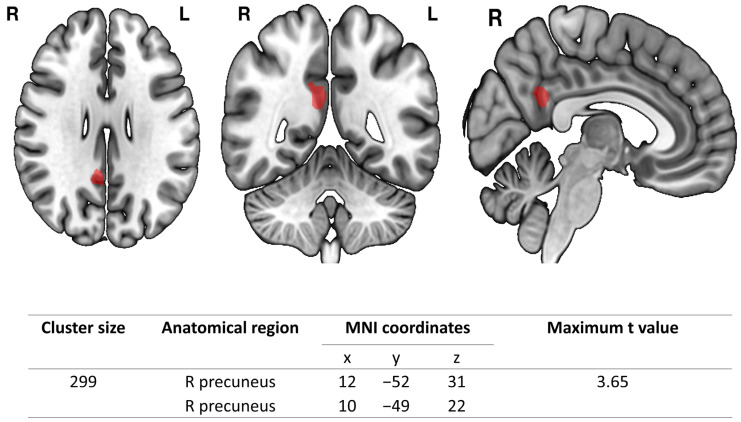
GMV difference between OSA and NC groups. Voxel-wise group comparisons of GMV between the OSA and NC groups show increased GMV of the right precuneus in the OSA group. There were no regions with decreased GMV in the OSA group compared to the NC group. Abbreviations: R: right; L: left; MNI: Montreal Neurological Institute.

The results of the voxel-wise whole brain analysis with full factorial design are demonstrated in Figure 2. OSA patients had significantly higher GMV in the right precuneus than the controls. There were no regions with significantly lower GMV in the OSA group as compared with controls.

### 3.4. Correlations between Plasma Biomarkers, Clinical Parameters, and Volumes of the Right Precuneus

**Figure 3:**
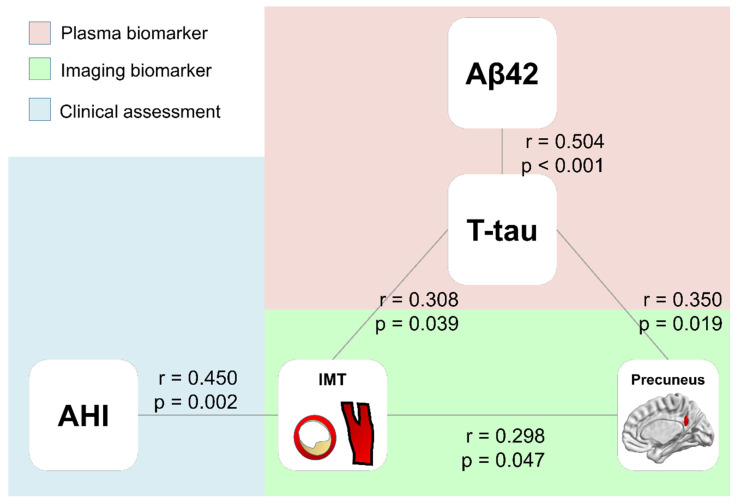
Correlations among plasma AD biomarkers, IMT, and GMV of the right precuneus. The T-tau plasma level is correlated with plasma levels of Aβ42, IMT of CCA, and GMV of the right precuneus. The IMT of CCA is correlated with GMV of the right precuneus and AHI.

The partial correlation analysis controlling for age and sex among plasma biomarkers, clinical parameters, and volumes of right precuneus are shown in Figure 3. The plasma T-tau level was positively correlated with plasma Aβ42, the IMT of the common carotid artery, and the GMV of the right precuneus. The IMT of the common carotid artery was also positively correlated with the apnea-hypopnea index (AHI) and the GMV of the right precuneus.

### 3.5. Correlations between Cognitive Function and Plasma Biomarkers, Clinical Parameters, and Volumes of Right Precuneus

Due to the exploratory nature of this study, correlation analysis between other parameters and NPT including digit span, orientation, information, comprehension, and drawing were conducted (Supplementary Table S1). None of the cognitive subtest showed significant correlation with plasma biomarkers, clinical parameters, and volume of right precuneus. The volume of right precuneus showed a trend for weak correlation with the information subtest score (r = 0.201; *p* = 0.111).

## 4. Discussion

For this study, we enrolled a group of OSA patients without associated cardiovascular or cerebral disorders, revealing three major findings. First, we identified elevated plasma levels of AD biomarkers in the group of OSA patients with normal cognition. Second, OSA patients had increased GMV in the right precuneus as compared to controls. Third, the level of plasma T-tau correlated with structural changes of the right precuneus and the IMT of the common carotid artery.

In contrast to many previous studies using the enzyme-linked immunosorbent assay (ELISA) method [21,22,23], in this study we used immunomagnetic reduction (IMR) technology for ultra-high-sensitivity detection (at pg/mL level) of plasma AD neuropathology biomarkers [24]. We found elevated levels of Aβ42 and T-tau in the OSA patients compared to NC. These findings are consistent with previous studies reporting increased CSF and blood amyloid and tau levels in OSA patients [5,25]. The plasma level of amyloid β is correlated with CSF amyloid β level and amyloid deposition in the brain [26], while the level of plasma tau correlates with CSF tau level [27]. The possible role and mechanism for peripheral amyloid β include the clearance of brain amyloid β into plasma via the blood-brain barrier (BBB) transporter [28], and blood-derived amyloid β crossing the BBB, which may trigger amyloid β production in the brain [29]. The plasma amyloid β may also reflect the compensatory clearance function from peripheral organs such as the liver, kidney, and gastrointestinal tract [30,31]. As our study found increased levels of AD neuropathology plasma biomarkers in the group of OSA patients without cognitive impairment, the observed increase in the plasma biomarkers may be interpreted as early changes that precede the development of cognitive impairment in OSA patients. However, future longitudinal studies are needed to confirm these temporal relationships.

Our investigation further revealed increased GMV in the precuneus of OSA patients. The precuneus is not only a key node in the default mode network at rest, but it also plays an important role in memory tasks [32]. Previous studies have shown that, as compared to controls, OSA patients exhibit an overactivation of the precuneus [33,34]. Meanwhile, previous studies have reported disparate findings with regards to structural changes in the brain of OSA patients. Some studies have reported increased GMV in OSA patients [34,35,36,37], while other studies have reported decreased GMV [12,38]. The discrepancies may be due to differing sample sizes, disease severity, or imaging modalities. In the present study and others [35,36], the enrollment of patients without cognitive impairment or patients with milder disease severity may have facilitated the detection of earlier stage brain structural changes.

Possible mechanisms for increased GMV include a compensatory effect, higher brain reserve, cerebral vasogenic edema related to hypoxia, or that the increased GMV may precede volume loss and hypometabolism [37,39,40]. Our previous study reported increased GMV at the precuneus, insula, and cerebellum, which were reversible after surgery in OSA patients who failed continuous positive airway pressure (CPAP) therapy [37], supporting the hypothesis that the increased GMV may be attributed to vasogenic edema caused by hypoxia. The correlation analysis showed no significant correlation between AHI and brain structural changes, which could be due to the fact that we enrolled patients with milder disease severity and in earlier stages.

The study identified a correlation between increased plasma T-tau level and increased GMV of the precuneus. This is consistent with a previous study showing colocalization of increased amyloid burden with increased GMV in the precuneus in a group of sleep-disordered breathing patients without cognitive impairment [41]. By using a multimodal imaging study including structural MRI, fluroine 18–labeled (^18^F), florbetapir PET, and ^18^F-fluorodeoxyglucose (FDG) PET scans, André C et al. reported increased amyloid burden, GMV, perfusion, and metabolism in the posterior cingulate gyrus and precuneus [41]. The correlation may be due to a common underlying pathophysiology. The intermittent hypoxia in OSA patients causes increased oxidative stress and neuroinflammation, which may cause both increased AD neuropathology and structural changes of the brain [37,42]. Furthermore, since we enrolled patients without cognitive impairment, the findings of a positive correlation between GMV and T-tau level may indicate early changes of the brain prior to atrophy, which may subsequently progress to irreversible structural deficit [37,43]. This information may be pertinent to the clinical setting, as timely intervention for OSA patients before the loss of reversibility in structural changes may result in better prognosis. Due to the exploratory nature of this study, we conducted correlation analysis on the digit span, orientation, information, comprehension, and drawing subtest. The results showed trend for positive correlation between the volume of the right precuneus and information subtest score without reaching statistical significance. The information subtest measures general information knowledge from the culture and is least affected by age [44]. Our result is in accordance with a previous study showing positive correlation between gray matter volume and information subtest in highly educated older adults [45]. In this study there was no significant correlation between cognitive function test scores and plasma AD biomarker, which may be due to the limited discriminating ability of the cognitive tests used in this study. Future studies using more sensitive neuropsychological assessment and the enrollment of patients with mild cognitive impairment or dementia may further elucidate their relationships.

OSA is associated with cardiovascular conditions such as hypertension, coronary artery disease, diabetes, metabolic syndrome, and cerebrovascular disease [8]. The relationship between increased carotid IMT and OSA has been reported by previous studies [9,10]. This correlation may be due to increased oxidative stress and inflammation exhibited in OSA patients [9,10]. The correlation among carotid IMT, plasma T-tau, and precuneus GMV identified in this study may be due to a common underlying mechanism; more specifically, the increased oxidative stress and inflammation can cause vascular damage and brain injury [8,37,42,46]. Alternatively, the vascular damage may lead to dysfunction of the BBB and hypoperfusion of the brain, resulting in the accumulation of tau and amyloid β [11]. Since APOE genotype is a major risk for AD and is strongly associated with cardiovascular diseases [47], future studies including this information with longitudinal design may elucidate the temporal relationship and the complex interaction among atherosclerotic disease, AD neuropathology, and brain structural changes in OSA patients.

## 5. Limitations

This study has several limitations. First, as cross-sectional data was analyzed, causal relationships cannot be inferred from correlations. Second, not all participants in the normal controls had PSG examinations; however, since subjects not having received a PSG examination had no complaints of sleep-related symptoms, the possibility of misclassification is likely not high. Third, there was indeed a significant difference between the sex of the two groups. Although sex was adjusted for in every statistical test, we could not exclude the effect of it on the results, such that the readers should interpret the results with caution. A larger study in the future that could perform separate analyses on both genders may clarify its interaction with brain structural changes and plasma AD biomarkers. Fourth, in this study we tested for T-tau to assess the general condition of neurodegeneration in OSA patients. A future study testing for phosphorylated tau (P-tau), which is a more specific marker for AD, may help to delineate the potential tractography of cognitive function in OSA patients. Lastly, there was no significant correlation between cognitive function and AD neuropathology or brain structural alterations, which may be partially due to the enrollment of only cognitively normal individuals in this study. The use of more sensitive cognitive tests for participants with normal cognition may elucidate their relationship in patients with normal cognition. Future longitudinal studies including patients with cognitive impairment may also provide more insight into the complex relationships between cognitive function, plasma AD biomarkers, and brain structural changes in OSA patients.

In conclusion, our study demonstrates that a group of OSA patients with preserved cognitive function exhibited elevated plasma levels of AD neuropathology biomarkers. The positive correlation between structural changes in the brain and the plasma level of T-tau in these patients may indicate early changes. The measurement of these biomarkers may aid in the early detection of OSA-associated morbidity and possible treatment planning for the prevention of irreversible neuronal damage and cognitive dysfunction.

## Figures and Tables

**Table 1:** Demographic data, plasma biomarker, clinical assessment, and neuro-psychological assessment data of patients with OSA and controls.

*Clinical Demographics and Plasma Biomarker*	OSA (n = 30)	Control (n = 34)	*p* Value
Age (year)	41.93 ± 1.65	43.21 ± 2.25	0.65
Sex (M, F)	27:3	17 ± 17	0.001 *
BMI	26.18 ± 0.52	24.83 ± 0.54	0.08
T-tau (pg/mL)	21.43 ± 0.55	18.27 ± 0.85	0.025 *
Aβ42 (pg/mL)	16.17 ± 0.12	15.37 ± 0.26	0.041 *
Aβ40 (pg/mL)	53.00 ± 0.90	54.61 ± 1.25	0.414
Aβ42/Aβ40	0.30 ± 0.04	0.29 ± 0.08	0.219
***Polysomnography parameters and*** ***IMT of CCA* ^#^**			
AHI	41.93 ± 4.33	2.68 ± 0.29	<0.001 *
ODI	32.75 ± 4.22	0.91 ± 0.21	<0.001 *
O_2_ < 90% (% per night)	8.47 ± 1.44	0.44 ± 0.28	<0.001 *
Average O_2_	95.00 ± 0.27	97.06 ± 0.19	<0.001 *
Snoring index	374.00 ± 33.82	233.62 ± 56.32	0.052
IMT	0.65 ± 0.12	0.54 ± 0.07	0.003 *
***Neuro-psychological assessments*** **Attention Function**			
Digit span	10.27 ± 2.57	11.53 ± 2.97	0.112
Attention	7.60 ± 0.72	7.74 ± 0.51	0.843
Orientation	17.93 ± 0.37	17.94 ± 0.24	0.332
**Executive Function**			
Digit symbol coding	10.87 ± 2.30	11.32 ± 2.04	0.943
Arithmetic	10.77 ± 2.24	10.53 ± 2.31	0.590
Abstract thinking	9.93 ± 1.26	10.03 ± 1.66	0.879
**Memory Function**			
Short-term memory	10.40 ± 1.34	10.33 ± 1.34	0.738
Long-term memory	9.87 ± 0.51	9.94 ± 0.34	0.510
Information	10.80 ± 3.02	11.21 ± 3.31	0.131
**Speech and Language**			
Comprehension	10.83 ± 2.45	11.12 ± 2.63	0.435
Language	9.85 ± 0.35	9.85 ± 0.36	0.693
Semantic fluency	8.80 ± 1.71	8.59 ± 1.78	0.884
**Visuospatial Function**			
Picture completion	11.50 ± 2.40	10.68 ± 2.67	0.504
Block design	11.37 ± 2.55	10.41 ± 3.00	0.555
Drawing	9.97 ± 0.18	9.94 ± 0.24	0.276

Abbreviations: OSA, obstructive sleep apnea; BMI, body mass index; Aβ, amyloid beta; AHI, apnea-hypopnea Index; ODI, oxygen desaturation Index; IMT, intima-media thickness; CCA, common carotid artery. Sex data were compared by Pearson chi-square test. Age and BMI data were compared by independent t test. The plasma biomarker, polysomnography parameters, and IMT of CCA were compared by analysis of covariance (ANCOVA) after controlling for age and sex. Neuro-psychological assessment data were compared by ANCOVA after controlling for age, sex, and education level. Data are presented as mean ± standard error of the mean for age, BMI, AD biomarker level, and polysomnography parameters. Data are presented as mean ± standard deviation for rest of the data. * *p* < 0.05. ^#^ Among 34 controls, data were only available in 17 subjects.

## Supplementary Materials

The following supporting information can be downloaded at: https://www.mdpi.com/article/10.3390/diagnostics12071522/s1, Table S1: Correlations between cognitive function and plasma biomarkers, clinical parameters, and volumes of right precuneus.
