# Supplementary material for: Increased Levels of Plasma Alzheimer’s Disease Biomarkers and Their Associations with Brain Structural Changes and Carotid Intima-Media Thickness in Cognitively Normal Obstructive Sleep Apnea Patients
Source: Diagnostics (Basel). 2022 Jun 22;12(7):1522. doi: 10.3390/diagnostics12071522 (PMC9324500; doi:10.3390/diagnostics12071522)
Supplement: Supplementary file 1 [file diagnostics-12-01522-s001.zip › diagnostics-1743452-supplementary.pdf]

Supplementary Table S1. Correlations between cognitive function and plasma biomarkers, clinical parameters, and volumes of right precuneus

		Digit span	Orientation	Information	Comprehension	Drawing
T-tau	r	-0.083	0.044	0.081	-0.054	0.126
	p	0.513	0.729	0.525	0.674	0.322
Aβ42	r	0.050	-0.031	0.157	0.176	0.139
	p	0.695	0.810	0.216	0.264	0.274
Aβ40	r	0.105	-0.108	-0.034	0.017	-0.074
	p	0.411	0.395	0.788	0.897	0.560
AHI	r	0.017	-0.118	-0.161	-0.149	-0.190
	p	0.910	0.431	0.279	0.319	0.200
IMT	r	0.214	0.074	0.182	-0.250	-0.139
	p	0.255	0.698	0.336	0.283	0.462
Precuneus volume	r	-0.024	0.168	0.201	0.165	0.078
	p	0.850	0.185	0.111	0.192	0.541
